# Segregation of Lexical and Sub-Lexical Reading Processes in the Left Perisylvian Cortex

**DOI:** 10.1371/journal.pone.0050665

**Published:** 2012-11-30

**Authors:** Franck-Emmanuel Roux, Jean-Baptiste Durand, Mélanie Jucla, Emilie Réhault, Marion Reddy, Jean-François Démonet

**Affiliations:** 1 UMR Unité 825, Faculté Paul Sabatier, IFR 96, Toulouse, France; 2 Pole Neurosciences, Centres Hospitalo-Universitaires, Toulouse, France; 3 Centre de Recherche Cerveau et Cognition, Toulouse, France; 4 U.R.I. Octogone-Lordat 4156, Université de Toulouse II, Toulouse, France; University of Barcelona, Spain

## Abstract

A fundamental issue in cognitive neuroscience is the existence of two major, sub-lexical and lexical, reading processes and their possible segregation in the left posterior perisylvian cortex. Using cortical electrostimulation mapping, we identified the cortical areas involved on reading either orthographically irregular words (lexical, “direct” process) or pronounceable pseudowords (sublexical, “indirect” process) in 14 right-handed neurosurgical patients while video-recording behavioral effects. Intraoperative neuronavigation system and Montreal Neurological Institute (MNI) stereotactic coordinates were used to identify the localization of stimulation sites. Fifty-one reading interference areas were found that affected either words (14 areas), or pseudo-words (11 areas), or both (26 areas). Forty-one (80%) corresponded to the impairment of the phonological level of reading processes. Reading processes involved discrete, highly localized perisylvian cortical areas with individual variability. MNI coordinates throughout the group exhibited a clear segregation according to the tested reading route; specific pseudo-word reading interferences were concentrated in a restricted inferior and anterior subpart of the left supramarginal gyrus (barycentre x = −68.1; y = −25.9; z = 30.2; Brodmann’s area 40) while specific word reading areas were located almost exclusively alongside the left superior temporal gyrus. Although half of the reading interferences found were nonspecific, the finding of specific lexical or sublexical interferences is new evidence that lexical and sublexical processes of reading could be partially supported by distinct cortical sub-regions despite their anatomical proximity. These data are in line with many brain activation studies that showed that left superior temporal and inferior parietal regions had a crucial role respectively in word and pseudoword reading and were core regions for dyslexia.

## Introduction

In neurocognitive models, word reading has been supposed to rely on two distinct processes that allow orthographic information to be matched to homologous phonological information. ‘Dual-route’ models proposed that words can be read either by an “indirect”, sub-lexical route, using grapheme to phoneme correspondence rules, or a “direct”, lexical route, in which words are directly recognized as lexicon members and associated with verbal semantic representations [1], [2], [3]. For instance, the sublexical route would be used to process new orthographic forms such as previously unknown words (i.e. rare words, foreign words, or experimental pseudo-words). The lexical route would be used to read common words, thought to be stored in an orthographic lexicon.

The dual-route models of reading originated from cases of dissociated reading disorders observed in patients with acquired dyslexia. Indeed, some alexic patients, presenting with phonological dyslexia, were found to name irregular words flawlessly but not pseudo-words [4], [5] whereas others, known as surface dyslexia patients, could process pseudo-words but not irregular words [1], [6], [7], [8]. According to lesion anatomy studies, different neural pathways and cortical areas could be associated with these lexical and sub-lexical processes. For instance, phonological acquired dyslexia has been linked to damage to left inferior-parietal [9] or left inferofrontal regions [10].

In brain imaging experiments, it has been shown that the phonological store (crucial to the efficient correspondence between orthographic sequences to their phonological counterparts) was localized in the supramarginal gyrus [11], [12] or in the left posterior superior temporal gyrus [13]. In a meta-analysis of 35 previous neuroimaging studies of reading, Jobard et al. [14] dissociated a lexicosemantic route involving the left basal temporal language area, the posterior part of the middle temporal gyrus, and the inferior frontal gyrus (pars triangularis) and a sublexical route involving left lateralized superior temporal areas, supramarginal gyrus, and the opercular part of inferior frontal gyrus. Nevertheless, controversies still exist about the anatomical substrates involved in lexical and sublexical reading routes [14], [15].

Direct cortical electrostimulation for neurosurgical cases offers researchers an invaluable technique to study human brain functions as it induces on-line transient inhibitory lesion-like effects on association areas. Stimulation-induced impairment of language performance during a given task indicates that the small area beneath the electrode is involved in the function elicited by the task. It has been shown that naming [16] or reading [17] are clinically relevant tasks during brain mapping. Our group previously studied some aspects of the organization of reading processes [18], [19], [20], [21], [22] showing that various reading scripts as musical score, Arabic numbers or alphabetic scripts from different languages could be anatomically segregated within left posterior Sylvian cortex. Another team, using words versus pseudo-words tasks, Simos et al. [23], [24] supported the existence of two different brain mechanisms for phonological processing in word reading; one in the posterior part of superior temporal gyrus subserving assembled phonology and a second, lexical mechanism that could be responsible for pronouncing words with rare print-to-sound correspondences depending on middle temporal gyrus. Beyond the scientific aspect, this finding could be important in practice when removing brain lesions within left posterior Sylvian region; it would imply that brain mappings should be performed using lexical and sublexical reading tasks.

In order to identify the perisylvian areas involved in reading and to spare them during surgery, cortical electrical stimulation mapping was used intraoperatively in 14 patients during the removal of brain tumours. Direct electrostimulation gave us opportunity to study the neural basis of reading processes in such crucial regions as the left perisylvian fissure and to address whether the two major, sub-lexical and lexical, reading processes are anatomically partially or entirely segregated within this region.

## Materials and Methods

### Patients

In this study, 14 patients (11 men - mean age: 42 year-old – Standard deviation: 18.6 - all right handed) underwent surgical resection of brain tumours or arteriovenous malformations (**Table 1**). In critical regions such as the posterior Sylvian fissure, electrostimulation was used in patients with deep seated cavernomas or metastasis to choose the best cortical approach (i.e. sparing functional areas). The surgical approach was modified according the functional data obtained by electrostimulation. Our surgical concept of tumour removal was to resect only tissue located within one centimeter of functional areas detected by electrostimulation. However, this principle approach was occasionally broken, if essentially necessary, to achieve tumour resection. All patients were French speakers and fulfilled the following criteria (1) had no language deficit pre-operatively, and (2) had a lesion located close to the left posterior Sylvian fissure. Language abilities were tested by one of us (ER, a speech therapist) in all subjects pre- and postoperatively, with standardized tests of visual naming, written and oral understanding, oral fluency, reading, dictation, repetition, written transcription, and object handling [25], [26] and handedness was assessed using the Edinburgh Inventory [27]. Patients with pre-operative language disorders were excluded from this study.

**Table 1:** Demographics and topography of explored brain regions - 14 patients.

Patient	Gender/Age/Occupation/Handedness (Edinburgh score)	Brain Lesion Treated
1	M/54/technician/Right Handed (+90)	High grade Glioma
2	M/23/student/Right Handed (+85)	High grade Glioma
3	M/22/student/Right Handed (+65)	Low grade Glioma
4	M/73/retired/Right Handed (+80)	Cavernoma
5	F/36/nurse/Right Handed (+65)	Cavernoma
6	M/14/student/Right Handed (+90)	Low Grade Glioma
7	M/32/technician/Right Handed (+70)	Low Grade Glioma
8	M/28/technician/Right Handed (+75)	Low Grade Glioma
9	F/48/social worker/Right Handed (+70)	Metastasis
10	M/34/computer engineer/Right Handed (+90)	Low Grade Glioma
11	M/62/retired/Right Handed (+80)	High grade Glioma
12	F/33/secretary/Right Handed (+80)	High grade Glioma
13	M/55/technician/Right Handed (+90)	High grade Glioma
14	M/70/notary/Right Handed (+90)	High grade Glioma

All the patients and their families gave their verbal and written informed consent to study their language areas by direct brain mapping. The National consultative committee of INSERM (Institut National de la Santé et de la Recherche Médicale) gave its approval for the storage of patients’ data and preservation of their anonymity. Data from these successive brain mappings were collected prospectively by the same team using the same protocol over throughout this three year-long period.

### Cortical Mapping: Tasks

We used 3 language tasks to map cortical areas: picture naming, word reading, pseudoword reading. Initially, the patients were asked to perform the naming task (visual naming task using drawings of various objects), followed by the word task and the pseudoword reading task. Each stimulation site was tested systematically with these 3 tasks. For each site, 4 or 5 word or pseudoword reading items were used in order to avoid “single” errors during brain mapping. Word reading considered of paramount importance by multiple testing to be certain that the tests were reproducible.

An assistant alerted the surgeon whenever performance impairments were induced by stimulation. When a functional site was found, it was marked by a sterile ticket of.25 cm^2^. Sub-cortical mapping was performed when appropriate but only with naming tasks.

To evaluate the use of each route, lexical and sublexical, we respectively used two sets of 36 stimuli: one of frequent French words and one of pronounceable pseudowords (see the full list of items in Table 2). The items were selected from a list of materials used in preoperative language tests. In order to maximize the use of the lexical route, the words selected were a) frequent words (median *freqlemlivres* = 32, Lexique 3, New, B. & Pallier, C., www.lexique.org); b) contained at least one irregular/inconsistent segment whose correct pronunciation cannot be obtained by using the most frequent segment-phoneme correspondence. Irregularities were due to different factors: irregular grapheme-phoneme mapping (se**c**ond, m**on**sieur, **oi**gnon), inconsistent rhyme orthography (n**erf**, g**ars**, n**et**) or loanwords (s**qu**are, pi**zz**a, f**oo**tb**all**). In order to maximize the use of the indirect, phonological route, without possible orthographic/semantic interaction, we selected pseudwords that could be pronounced in French but that do not have close orthographic neighbours. In order to compare results in word and pseudoword reading, we paired the stimuli for length (number of letter and syllable) and bigram frequency (see Table 3 for a statistical description of the material used).

**Table 2:** List of words and pseudowords used in this study.

Words	Pseudowords
deuxième	moube
nerf	butiro
chaos	dasul
sculpture	adrile
choix	écine
second	tople
longtemps	birzouko
gars	cande
square	tribul
oignon	voral
respect	durche
franc	atrul
orgueil	dipulo
football	énoure
gentil	nacide
violemment	toupre
net	nochir
aiguille	sinope
patience	nurin
flux	afnur
banc	cipre
pizza	jamik
chronique	pudiro
hall	covulta
oeil	égibe
maximum	dripul
août	chulo
examen	filchon
six	cuifle
orchestre	saille
ville	pitode
dessous	prapou
poêle	bultir
choeur	pogide
monsieur	simade
poing	tanepi

**Table 3:** Comparative description of words and non words lists.

Items	Number of letters *(SD)*	Number of syllables *(SD)*	Mean bigram frequency *(SD)*
Words	5,75 *(0,7)*	1,9 *(0,6)*	2211 *(779)*
Non words	6 *(1,9)*	1,6 *(0,6)*	2455 *(1218)*
*p-value*	*0,74*	*0,07*	*0,32*

The task for word reading was performed first, and, after completion, patients performed the pseudo-word reading task. We chose orthographically irregular French words and phonologically legal pseudowords to contrast two modes of accessing output phonology. Reading irregular words elicits access to phonological output lexicon and assembled phonological representations. Pseudoword reading requires sub-lexical orthography-to-phonology mapping and segmented phonological processing [28].

### Cortical Mapping: Procedure

As described in previous studies [19], the cortex was directly stimulated using the bipolar electrode of the “Nimbus” cortical stimulator (1 mm wide electrodes separated by 6 mm: Newmedic^®^, Toulouse, France)**.** The current amplitude was started at 2 mA, and progressively increased by 1 mA, using biphasic square wave pulses of 1 ms at 60 Hz. Care was taken to avoid electrical diffusion and afterdischarges by stimulating under the level of stimulation generating afterdischarges. So, intraoperative cortical stimulation was used to localize the areas of the functional cortex after determination of the afterdischarge threshold roughly one second by electrocorticography.

### Accuracy of Electrostimulation Mapping

Electrostimulation is the oldest technique of brain mapping [16]. The main advantage of this technique is its high level of accuracy [29]. We used a low stimulation frequency known to activate preferentially cortical local cells [30] or afferent inputs of the stimulated area. The peak current density is localized in a small spherical region around the bipolar electrodes [31]. Indeed, the signal remains tangentially located in the immediate vicinity of the electrode which deters the cortical functions located in less than 1 cm^2^ under the probes [29]. During stimulation, we often observe that the displacement of the electrode to an adjacent region located 1 cm apart from the initial area results in strikingly different effects.

### Extent and Localization of Stimulated Sites

Stimulation was guided by a neuronavigational system, with 3D reconstructions of the brain (Stealth Station, Sofamor Danek, Surgical navigation technologies, Broomfield, CO, USA) to localize brain gyri. Each 3D brain volume was normalized in the MNI space, and parameters were used to obtain normalized coordinates from stimulation site locations, which were intraoperatively visualized and positioned on 3D original images provided by a neuronavigation software (Medtronic, Minneapolis, MN). The sites of cortical stimulation were 1 cm apart. The cortical surface tested was divided into several squares of 1 cm^2^. The extent and the number of cortical stimulation sites varied from one patient to another depending on the size of craniotomy.

### Conditions of Validation

Criteria for validation of interference sites can be summarized in 3 points:

1. We think that video recording is crucial to data monitoring so that observations can be validated or discarded from the analysis. All patients of this series had their cortical mapping procedures video recorded. Video recordings were especially helpful to improve postoperative analysis of type of interference (phonemic substitution, jargon, etc…) obtained during reading tasks.
2. To be accepted as “word reading site” or “pseudo word reading site”, the identified interference sites were meticulously tested at least 3 times (not consecutively). Cortical sites showing no 3 reproducible task interferences were not included in this study. In cases of ambiguity, a site could be tested more than 3 times.
3. Finally, it must be emphasized that, when we qualified a reading site as “pseudo word-specific reading” it meant that neither naming nor word reading interferences were found in this area. We could not exclude, however, that this site defined as “specific” may lead to stimulation interference for other functions not tested in this study.

## Results

No general complications were noted in this series during operation. Current intensity that did not evoke afterdischarges ranged from 4.1 to 5.6 mA in this study. None of the patients had generalized seizures intra-operatively. We found reading interferences in all patients (3.6 reading interference per patient; Standard deviation: 2.3) but one (patient 14). Overall, a total of 51 reading interference sites were detected, common to both reading tasks (26 sites) or specific to one reading task (25 sites). These interference sites were detected in small cortical patches of 1, 2 or 3 cm^2^. Individualized reading maps were generated in terms of number, size, type and localization of reading interference sites.

### Number and Size of Reading Interference Areas

**Figure 1:**
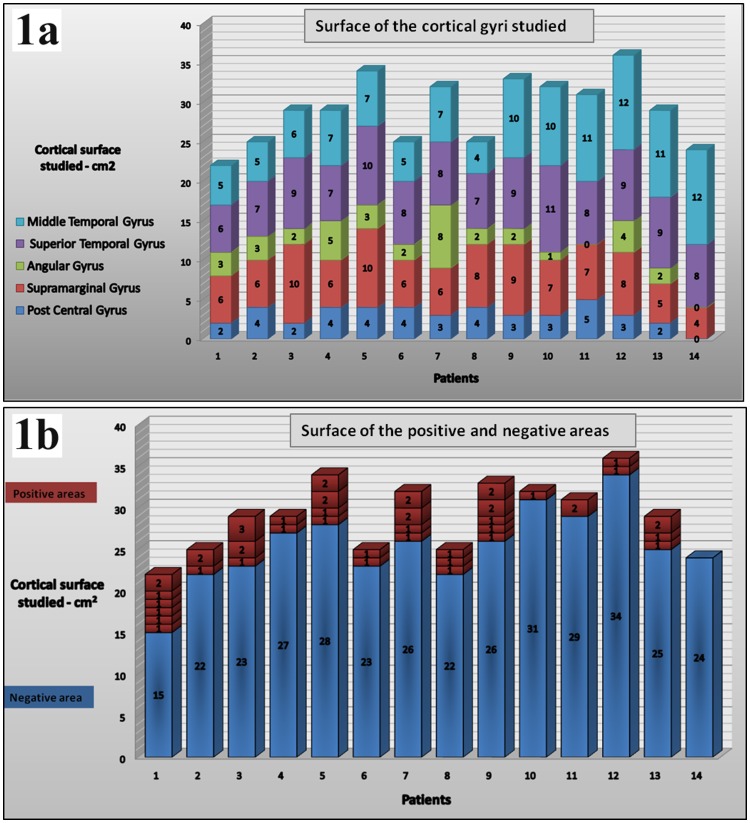
Number and size of reading interference areas. Figure 1a shows in each patient the surface of the cortical gyri studied with some variations according to the tumor location and size of bone flap. Figure 1b illustrates the variability of the number and surface of the positive reading areas among this group of patient. A majority of positive areas were discrete (1 cm^2^) cortical areas; adjacent sites (2 cm^2^) found to be both involved in reading was observed 11 times, and only in a single case did we find a positive area of 3 cm^2^.

Group analysis on 14 patients showed that the mean area of cortical surface tested and calculated on operative 3D MRI reconstructions was 29 cm^2^ (range, 22 to 36 cm^2^ – Standard deviation, 4.24). Interferences for reading were observed for stimulation of discrete, highly localized patches of cortex. Twenty-six areas were “single” sites (i.e. sites occupying a surface of 1 cm^2^ with intervening areas evoking no reading interferences). The margins of these sites were distinct, the displacement of the electrode into an adjacent cortical area located in the same gyrus producing no reading interference. Reading interferences found varied from 0 to 7 (mean: 3.3 interferences per subject). The range of the cortex involved in word and pseudo-word reading routes for each patient was 3.64 cm^2^ (range, 0 cm^2^ to 7 cm^2^– standard deviation, 2.34). Neural structures found by electrostimulation and involved in reading occupied only 12.5% of the surface tested. **Figure 1a** details the surface of the cortical gyri studied in each patient and **Figure 1b** illustrates the variability of the number and surface of the positive reading areas found.

### Type and Localization of Interference of the Cortex Areas Involved in Reading

**Table 4** summarizes the type and the localization of interference found during reading mapping by direct stimulation in the 13 patients with positive results. Results of stimulations were divided in: 1) common interference areas for word and non word reading and 2) specific interference areas for either word or pseudo word reading. Over 51 reading interference sites, 26 common interference sites for word and pseudo word reading were found. Fourteen specific word interference areas and 11 specific pseudo-word interferences were detected. Interferences specific to pseudo words were detected in 6 patients (patients 1, 2, 5, 6, 8, and 13). Interferences specific to words were found in another group of 6 patients (patients 3, 5, 7, 8, 9, and 13) with partial overlap between the two groups, i.e. patients 5, 8 and 13 showed each interference sites specific to either words or pseudo-words. In cortical areas where word and pseudo-word reading interferences were observed (common areas), types of reading disorders observed during electrostimulation were similar for both tasks. The 51 positive interferences either elicited by pseudo-word and word reading tasks, 41 (80%) corresponded clearly to the impairment of the phonological level of reading processes (i.e. electrostimulation elicited jargon, neologism, substitution of syllabes, syllabation). Other interferences were reading arrest (blockade) or various phenomena such as hesitations or auto corrections.

**Table 4:** Intraoperative Talairach’s coordinates (MNI) of sites showing interference upon stimulation in the explored group of 14 patients.

Patient	MNI coordinates of detected interference area			Word, non-word or common interference	Type of reading interference	Anatomical Area/BA
	X	Y	Z			
**1**	−70	−47	5	Common	Auto-corrections	Superior Temporal Gyrus/22
**1**	−70	−36	2	Common	Hesitation	Middle Temporal Gyrus/22
**1**	−70	−24	−1	Common	Letter –level errors	Superior Temporal Gyrus/22
**1**	−68	−8	−3	Common	Blockade	Superior Temporal Gyrus/21
**1**	−70	−20	12	Common	Blockade	Superior Temporal Gyrus/42
**1**	−68	−14	11	Non-Word	Letter –level errors	PostCentral Gyrus/43
**1**	−69	−14	16	Non-Word	Slowing/Syllabations	PostCentral Gyrus/43
**2**	−66	−40	27	Common	Letter –level errors	Supramarginal Gyrus/40
**2**	−65	−36	37	Non-Word	Letter –level errors	Supramarginal Gyrus/40
**2**	−61	−36	52	Common	Slowing/syllabation	PostCentral Gyrus/40
**3**	−68	−32	8	Common	Blockade	Superior Temporal Gyrus/42
**3**	−68	−45	9	Word	Neologism/jargon	Superior Temporal Gyrus/22
**3**	−68	−37	20	Word	Neologism/jargon	Superior Temporal Gyrus/22
**3**	−64	−48	26	Word	Letter –level errors	Supramarginal Gyrus/40
**3**	−65	−37	29	Common	Articulation/voice alteration	Supramarginal Gyrus/40
**3**	−65	−31	31	Word	Neologism/jargon	Supramarginal Gyrus/40
**4**	−68	−39	30	Common	Blockade	Supramarginal Gyrus/40
**4**	−70	−48	−10	Common	Blockade	Middle Temporal Gyrus/21
**5**	−65	−32	43	Non-Word	Letter –level errors	Supramarginal Gyrus/40
**5**	−68	−31	36	Non-Word	Letter –level errors	Supramarginal Gyrus/40
**5**	−68	−23	36	Non-Word	Slowing/syllabations	PostCentral Gyrus/2
**5**	−71	−25	6	Word	Letter –level errors	Superior Temporal Gyrus/42
**5**	−66	−42	40	Word	Letter –level errors	Supramarginal Gyrus/40
**5**	−65	−46	35	Word	Neologism/jargon	Supramarginal Gyrus/40
**6**	−68	−36	32	Non-Word	Letter –level errors	Supramarginal Gyrus/40
**6**	−70	−26	31	Non-Word	Letter –level errors	Supramarginal Gyrus/40
**7**	−65	−36	42	Common	Letter –level errors	Supramarginal Gyrus/40
**7**	−67	−29	42	Common	Letter –level errors	Supramarginal Gyrus/40
**7**	−69	−21	33	Common	Slowing/syllabation	PostCentral Gyrus/2
**7**	−70	−25	22	Common	Autocorrections	PostCentral Gyrus/40
**7**	−71	−36	21	Word	Neologism/jargon	Superior Temporal Gyrus/22
**7**	−71	−39	27	Word	Word-level	Supramarginal Gyrus/40
**8**	−67	−36	30	Word	Slowing/syllabation	Supramarginal Gyrus/40
**8**	−65	−49	17	Common	Neologism/jargon	Superior Temporal Gyrus/22
**8**	−68	−21	36	Non-Word	Slowing/syllabation	PostCentral Gyrus/3
**9**	−68	−30	24	Word	Blockade	Supramarginal Gyrus/40
**9**	−65	−17	26	Common	Letter –level errors	PostCentral Gyrus/43
**9**	−70	−12	20	Common	Letter –level errors	PostCentral Gyrus/43
**9**	−68	−6	19	Common	Letter –level errors	PostCentral Gyrus/43
**9**	−72	−20	5	Word	Neologism/jargon	Superior Temporal Gyrus/22
**9**	−71	−13	4	Word	Slowing/syllabation	Superior Temporal Gyrus/22
**9**	−62	0	−6	Common	Neologism/jargon	Superior Temporal Gyrus/22
**10**	−68	−18	26	Common	Letter –level errors	PostCentral Gyrus/2
**11**	−68	−23	36	Common	Neologism/jargon	PostCentral Gyrus/2
**11**	−65	−24	41	Common	Letter –level errors	PostCentral Gyrus/2
**12**	−69	−43	22	Common	Letter –level errors	Supramarginal Gyrus/40
**12**	−69	−49	12	Common	Letter –level errors	Superior Temporal Gyrus/22
**13**	−72	−20	9	Common	Letter –level errors	Superior Temporal Gyrus/42
**13**	−69	−5	1	Word	Slowing/syllabation	Superior Temporal Gyrus/22
**13**	−70	−26	23	Non-Word	Letter –level errors	Supramarginal Gyrus/40
**13**	−70	−26	31	Non-Word	Letter –level errors	Supramarginal Gyrus/40
**14**	No interference detected					

BA: Brodmann’s areas. Each site had a cortical surface of 1 cm^2^. Sites in dotted square are adjacent sites (2 or 3 cm^2^).

**Figure 2:**
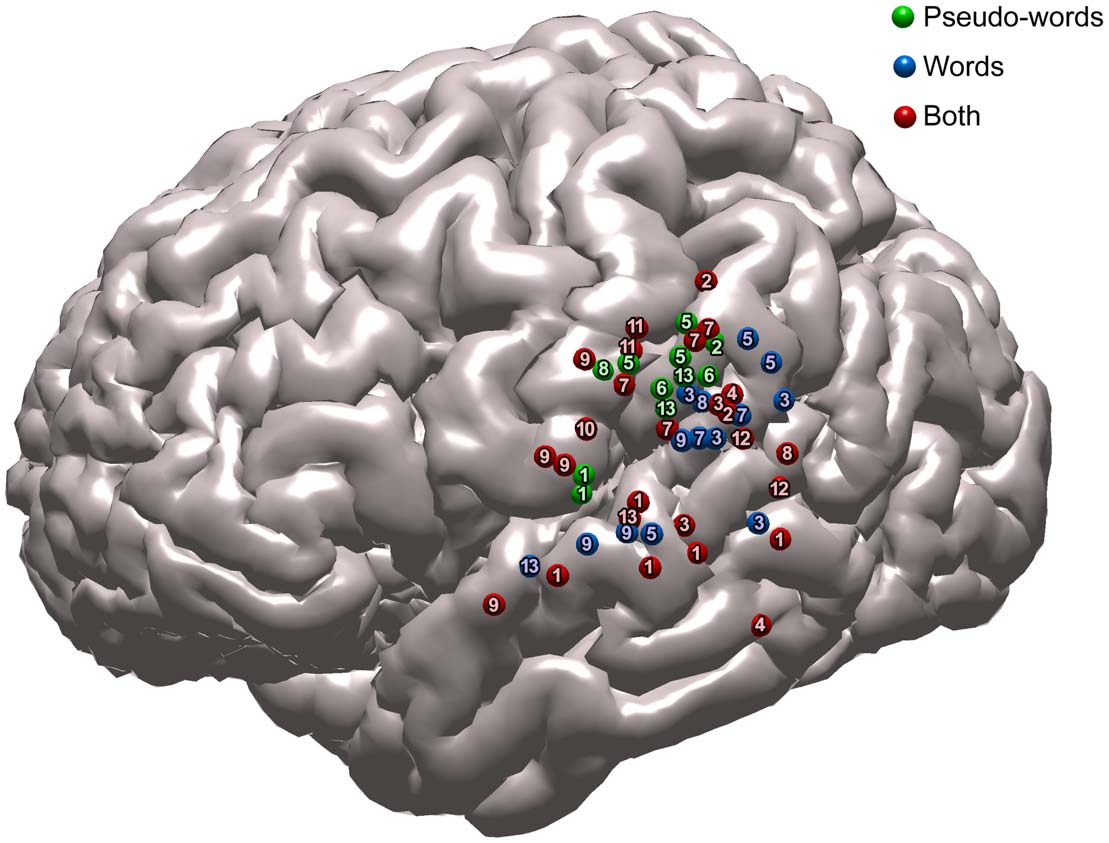
Localization of the word and pseudo word sites. Figure 2 shows the localization of word and pseudo word reading stimulation sites positioned in the standard normalized Montreal Neurological Institute (MNI) space (listed in Table 3 - Numbers inside the dots refer to patient number listed in this table). Overall, 26 common words and pseudo word reading interferences (red) were found in different regions of posterior Sylvian fissure. Fourteen specific word reading (blue) and 11 specific pseudo word reading were detected (green). Group analysis demonstrated that specific word or pseudo word reading areas exhibited a clear somatotopy, pseudo-word reading interferences being located in the anterior part of the supramarginal gyrus (barycentre: x = −68.1; y = −25.9; z = 30.2; Brodmann’s area 40) and word reading areas in the temporal gyrus (barycentre: x = −68.3; y = −32.4; z = 19.9 Brodmann’s area 42).

**Figure 3:**
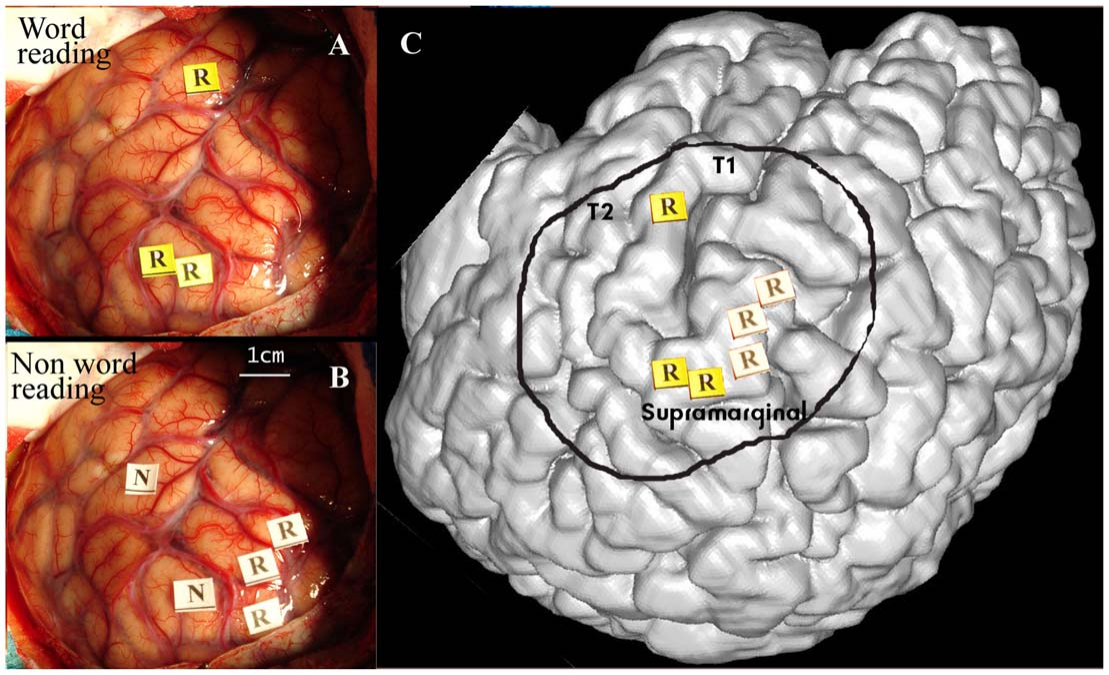
Example of cortical mapping in patient 5. Figure 3 shows in patient 5 the results of word (R yellow) and pseudo word reading (R white) mapping in a patient who had an arteriovenous malformation within the posterior part of the Sylvian fissure. Word reading (A) involved the posterior part of supramarginal gyrus and T1 gyrus whereas pseudo word reading (B) involved anterior part of supramarginal gyrus. To improve the understanding of the intraoperative pictures, cortical sites producing no reading interference (Negative area = N) were not systematically noted by a sterile ticket. These sites were extremely localized in small areas of the cortex. In this case, as seen in the 3D reconstruction of patient’s brain (in C) strict segregation of the areas involved in word or pseudo-word reading was found.

**Figure 2** summarizes the results of reading mapping by direct stimulation obtained in the patients. In group analysis, specific word or pseudo word reading areas exhibited a clear topographical segregation; pseudoword reading interferences were located in the anterior part of the supramarginal gyrus or the posterior part of postcentral gyrus (Brodmann’s area 3, 40 or 43) and word reading areas in the superior temporal gyrus (Brodmann’s area 22 and 42) or a more posterior part of the supramarginal gyrus. Common interferences for word and pseudo word reading were mainly observed in superior temporal gyrus and anterior part of the supramarginal gyrus. In the 3 patients (Patients 5, 8, and 13) showing interferences of each of the two reading processes, the specific sites were clearly segregated in anatomical terms; pseudoword reading interferences were concentrated in the inferior/anterior part of the left supramarginal gyrus, while word interference sites were more distributed, being found either in the posterior part of the supramarginal gyrus or the superior temporal gyrus (**Figure 3**).

## Discussion

In this study, we found that reading processes involved discrete, highly localized left perisylvian cortical areas with individual variability. Partial segregation of the pseudo-word and word reading routes was detected, specific pseudo-word reading interferences being concentrated in a restricted inferior and anterior subpart of the left supramarginal gyrus while specific word reading areas were located almost exclusively alongside the left superior temporal gyrus. Despite their anatomical proximity, lexical and sublexical processes of reading could be supported by distinct cortical sub-regions, at least partially. Moreover, the literature on brain-damaged patients suggests that these pathways are not fully independent but instead interact [32]. It is worth to note that pseudo-word/word anatomical segregation has also been described in left occipitotemporal cortex [7], [8], [22], [33], [34], [35]. This partial dissociation between pseudo-word reading and irregular word reading suggests that the left inferior-parietal cortex could be more involved in pseudo-word reading than the left temporal cortex that is thought to relate to lexical long-term memory and therefore might be more involved in the processing of irregular words. Patients with acquired phonological dyslexia are poor at reading pseudo-words, whereas their word reading is relatively spared [5]. This could possibly result from impairment in graphemic-phonologic conversion, which is thought to depend mainly on functions of the left inferior-parietal [9] and inferior frontal regions [10].

However, the numerous neuroimaging studies [21], [36], [37], [38] on this topic yield rather controversial conclusions in establishing the organization of lexical and sublexical processes within the posterior Sylvian region [14], [15]. Main difficulties arose from the fact that words and pseudowords differ in more than one way (length, familiarity, access to semantics and phonological decoding) and because differences in activation were often relative within detected areas [14], [15]. The supramarginal gyrus seems especially involved in studies dealing with pseudo-words or unfamiliar letter combinations [39]. A recent study [35], taking into account many psycholinguistic parameters that could be responsible for differential activations during word versus pseudo-word reading, put forward specific brain regions involved in direct orthography to phonology mapping (including the left supramarginal gyrus) or lexical/semantic processing (left middle and inferior temporal regions, bilateral angular gyri). Other authors have challenged these conclusions, pointing the fact that, overall, pseudo-words and real-words recruit the same neural areas [15], [21], [40], [41] maybe with some differences in terms of spatiotemporal dynamic of activation between the two word classes [41]. Other claimed that the very small number of subjects of some case report studies do not allowed reaching clear conclusions [15].

Regarding the connectivity between these areas, the reading process could be mediated by the temporo-parietal section of the arcuate fasciculus from the inferior temporal lobe to inferior-parietal region [42]. The superior longitudinal fasciculus could also convey reading information from inferior-parietal region to dorsal prefrontal cortex [21]. Overall, both ventral and dorsal pathways could process frequent regular words [1]. Levy et al., investigated the use of these two pathways in an effective connectivity study during word or pseudo-word reading [22]. They showed that both routes could be involved in processing both types of stimuli and that using the appropriate route was predictive of better reading performance. The use of both ventral and dorsal pathways in word and pseudo-word reading could partly explain the composite reading maps generated within the left perisylvian cortex in our population.

In this study, we found that pseudo-word and word reading could involve discrete, highly localized (often limited to small neuronal patches less than 1 cm^2^) perisylvian cortical areas with some individual variability. Composite and individual reading maps generated in this study may be linked to cytoarchitectonic studies showing high variability of cortical microstructures within the inferior parietal lobe [43]. Other hypotheses could be raised to explain this variability such as the influence of epileptic activity (although none of our patients had chronic epilepsy) as well as the individual variability in the type of brain disease treated; slow-growing tumors (5 patients in this study) in areas which could lead to a local potential re-organization of higher cortical functions.

This variability did not prevent us to observe that not only the left perisylvian cortex was crucial to the phonology-level coding of verbal stimuli but that both sublexical and lexical reading routes could be anatomically segregated within this region. Although, at the individual level, an overlap may exist in this region between pseudo-word and word processing, group analysis reveals a certain degree of topographical segregation between sub-lexical and lexical types of language processes. The grapho-phonological conversion linked to the sublexical reading process seems to involve the anterior part of the left supramarginal gyrus. This is in line with our previous works describing the involvement of the supramarginal gyrus in phonological processing of sentence reading [19] and with the existence of a spatial segregation of the phonological process for the 2 different types of script (alphabetic versus arabic number) in this gyrus [18]. This study and the current psycholinguistic models of reading also suggest that the superior temporal gyrus and the more posterior part of the supramarginal gyrus are involved in the phonological output level of the lexical reading process. Indeed, irregular word reading interferences found in this region stand at the border of the angular gyrus (BA 39) whose specific implication in the lexical route of reading, has been frequently described [13], [44], although its lexical and/or semantic specificity is still controversial [14]. These data are congruent with 2 recent meta-analysis on brain activation studies on normal reading and dyslexia [45], [46]. In a meta-analysis on functional imaging data of reading, Cattinelli et al. claimed that reading involves a pseudoword-related network in the left inferior parietal cortex and a word-related network in the left temporal lobe [45]. Analyzing 17 neuroimaging studies on dyslexia, Richlan et al. found that these left inferior parietal and superior temporal regions were underactivated in dyslexic readers [46]. Moreover, white matter abnormalities were also detected in developmental dyslexic in these regions [47].

### Conclusion

Data from literature suggest that segregation between both reading routes could involve the left lateral superior temporal cortex with the left ventral inferior frontal gyrus for the lexicosemantic route and the postero-superior temporal and anterior inferior parietal cortices for a non-semantic phonological decoding route. As shown in this study, these pathways can overlap in the left posterior Sylvian region but also dissociate. With the previous electrostimulation studies on the same topic [23], [24], these findings could be clinically relevant. The cortical and subcortical individual variability seen during surgery is an essential factor and plays a significant role in map reading and tumour resection in each patient. Nevertheless, in the frame of this study, we could not show that individual word and non word testing during surgery were essential for the functional prognosis of patients (i.e. is removing of those specific areas during a tumor resection, lead to significative and permanent language disturbances?). However, we believe that patients with tumors or arteriovenous malformations in this left posterior Sylvian region could be tested with different reading tasks in order to effectively preserve, as much as possible, these functional areas. Finally, we hypothesized that the individual pattern of the organization of reading within the posterior Sylvian fissure and the discrete spatial segregation of these cortical neuronal structures sometimes separated by less than 1 cm could explain the difficulties in reaching a consensus within the numerous brain imaging studies that have been devoted to reading.
